# Quantification of chitooligosaccharides by FACE method: Determination of combinatory effects of mouse chitinases

**DOI:** 10.1016/j.mex.2020.100881

**Published:** 2020-04-18

**Authors:** Masahiro Kimura, Takatoshi Umeyama, Satoshi Wakita, Kazuaki Okawa, Masayoshi Sakaguchi, Vaclav Matoska, Peter O. Bauer, Fumitaka Oyama

**Affiliations:** a Department of Chemistry and Life Science, Kogakuin University, Hachioji, Tokyo, Japan; b Research Fellow of Japan Society for the Promotion of Science (DC2), Koujimachi, Chiyoda-ku, Tokyo 102-0083 Japan; c Laboratory of Molecular Diagnostics, Department of Clinical Biochemistry, Hematology and Immunology, Homolka Hospital, Roentgenova 37/2, Prague 150 00, Czech Republic; d Bioinova Ltd., Videnska 1083, Prague 142 20, Czech Republic

**Keywords:** Fluorophore-assisted carbohydrate electrophoresis, Chitinase, Chitooligosaccharides, Chitin degradation, Quantitative analysis

## Abstract

Fluorophore-assisted carbohydrate electrophoresis (FACE) enables detection and quantification of degradation products from artificial and natural chitin substrates such as 4-NP-(GlcNAc)_2_, (GlcNAc)_4_ and colloidal chitin. The FACE method has been improved by our group for analysis of chitooligosaccharides in the presence of several buffer systems commonly used in the biochemical evaluation of chitinolytic activities of enzymes at pH 2.0–8.0. FACE is a very sensitive technique detecting picomolar amounts of molecules. We optimized the detection conditions as follows: exposure type, precision; sensitivity, high resolution; exposure time, 5 s. We evaluated the (GlcNAc)_2_ levels using a standard curve that allows chitooligosaccharides quantification at up to 10 nmol amounts. Using the method presented here, the chitinolytic properties of different chitinases can be compared directly. *Serratia* chitinase A (ChiA) and chitinase B (ChiB), two well-studied bacterial chitinases, have been shown by HPLC to have a synergistic effect on the chitin degradation rate. Using the FACE method, we determined the combinatory effects of mouse chitotriosidase (Chit1) and acidic mammalian chitinase (AMCase) in natural chitin substrates processing.

• FACE is a simple and quantitative method.
• Our improved procedure enables the quantification of chitooligosaccharides produced by chitinases at pH 2.0–8.0.
• FACE is able to quantify chitooligosaccharides at up to 10 nmol amounts.

## Highlights

• FACE is a simple, qualitative and quantitative method.
• The standard curve warrants quantification of chitooligosaccharides of up to 10 nmol regardless on used buffer system.
• Our improved FACE method enable us to quantify chitooligosaccharides produced by chitinases at pH 2.0–8.0.
• Determination of the combinatory effects of the Chit1 and AMCase using the FACE method.

## Graphical abstract

**Figure:**
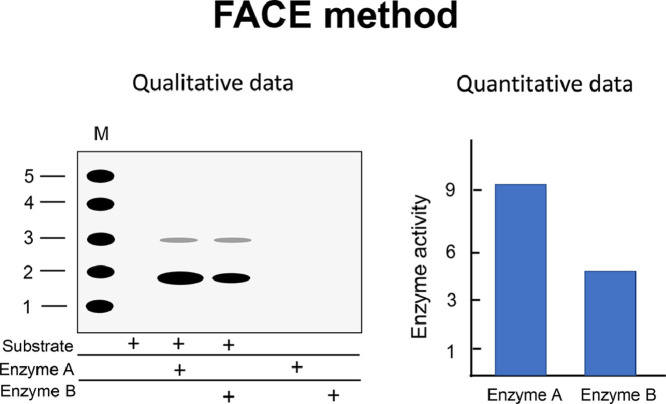

**Specifications table**

**Table:** 

Subject Area:	• Biochemistry, Genetics and Molecular Biology
More specific subject area:	Enzymology
Method name:	Improved FACE method
Name and reference of original method:	FACE method
	*P. Jackson, The use of polyacrylamide-gel electrophoresis for the high-resolution separation of reducing saccharides labeled with the fluorophore 8-aminonaphthalene-1,3,6-trisulphonic acid. Detection of picomolar quantities by an imaging system based on a cooled charge-coupled device, Biochem. J. 270(3) (1990) 705-13.*
Resource availability:	8-aminonaphthalene-1,3,6-trisulphonic acid (ANTS, Thermo Fisher Scientific, Waltham, MA, USA), Centrifugal Concentrator CC-105 (TOMY, Tokyo, Japan), Luminescent Image Analyzer (ImageQuant LAS 4000, GE Healthcare),

## Method details

### Background

Fluorophore-assisted carbohydrate electrophoresis (FACE) is a method based on fluorescent labeling of the reducing ends of oligosaccharides. It is a very sensitive technique (pmol levels) as compared to high-performance liquid chromatography (HPLC) and nuclear magnetic resonance (NMR) spectrometry and is often used for detection of very low oligosaccharide quantities [1,2].

ANTS and 2-aminoacridone (AMAC) have been used for fluorescent labeling of the reducing end of oligosaccharides. Jackson (1990) has shown that ANTS can label the reducing end of 35 different oligosaccharides [1]. Recently, we have found that the by-products in the fluoresceinated reaction were formed by labeling at pH >5 and introduced a pre-acidification step to suppress such products formations [3]. We hypothesized that the improved Jackson method using ANTS could be suitable for chitooligosaccharides labeling. Using this method, we determined the combinatory effects of mouse chitinase in natural chitin substrates processing.

### Fluorescent labeling

1. The reaction solution was quickly freeze-dried under vacuum using Centrifugal Concentrator CC-105 (TOMY, Tokyo, Japan).
2. To the freeze-dried samples, 5 µL of 0.2 M 8-aminonaphthalene-1,3,6-trisulphonic acid (ANTS, Invitrogen, Carlsbad, CA, USA) in acetic acid/water (3:17, v/v), 5 µL of 1.0 M NaCNBH_3_ in dimethyl sulfoxide (DMSO) and 5 µL of 17.5 M acetic acid was added.
3. The samples were incubated at 37 °C for 16 h.
4. The solution was then neutralized by 15 µL of 1 M NaOH, followed by 15 µL of loading buffer addition without SDS, 2-mercaptoethanol and bromophenol blue [4].

### Separation and quantification

1. The 40% polyacrylamide gel was prepared according to the composition in Table 1.

**Table 1:** Composition of the gel.

Separating gel (Two pieces of gel)	
stock solutions	volume
60% acrylamide/1% bis-acrylamide	10 mL
1 M Tris–HCl (pH 8.8)	5 mL
10% ammonium persulfate	50 μL
TEMED	10 μL

Stacking gel (Two pieces of gel)	
stock solutions	volume
H_2_O	5.8 mL
30% acrylamide	1.7 mL
1% bis-acrylamide	1.3 mL
1 M Tris–HCl (pH 6.8)	1.3 mL
10% ammonium persulfate	50 μL
TEMED	10 μL
2. The samples were separated by polyacrylamide gel electrophoresis (PAGE), as described previously [1,3].
3. The electrophoresis plate was 106 mm wide and 100 mm high (Mini gel slab electrophoresis device; Oriental Instruments, Sagamihara, Kanagawa, Japan).
4. The electrophoresis voltage was set at 150 V for the stacking gel and 150–250 V for the separating gel.
5. When the above apparatus was used, the electrophoresis time was 4 h.
6. The samples were quantified using the Luminescent Image Analyzer (ImageQuant LAS 4000, GE Healthcare), according to the manufacturer's instructions (Transillumination at 312 nm. Exposure conditions were fixed as follows: exposure type, precision; sensitivity, high resolution; exposure time, 5 s).
7. The samples were quantified using Analysis Toolbox according to the Luminescent Image Analyzer manual.

## Method validation

### Enzyme reaction

**Fig. 1:**
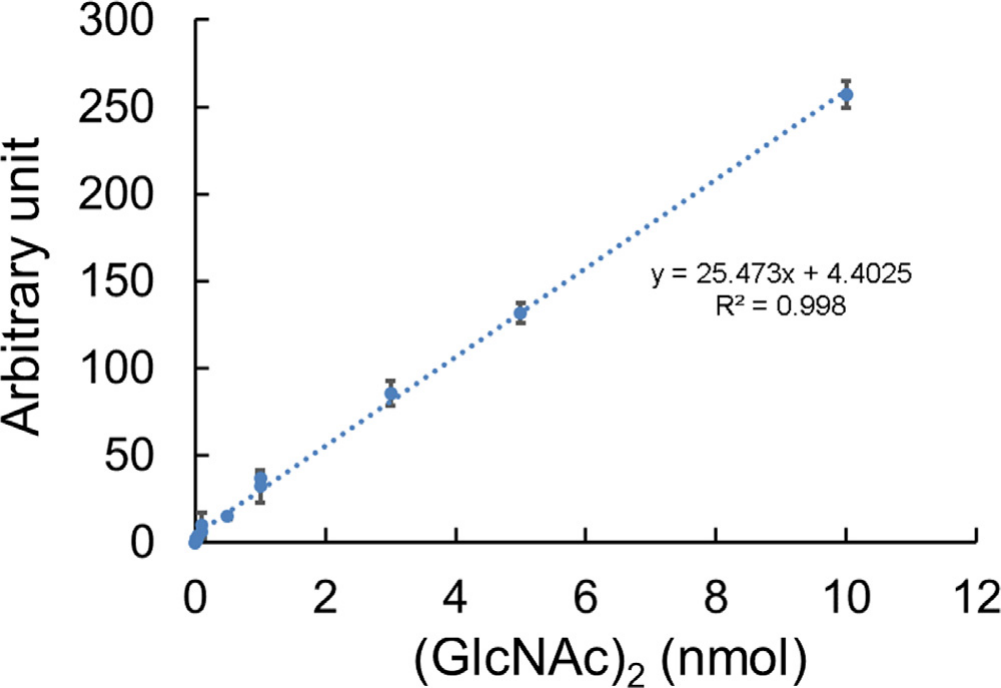
The standard curve warrants quantification of (GlcNAc)_2_ of up to 10 nmol. There was a linear relationship between the (GlcNAc)_2_ amount and the fluorescence intensity. Data were acquired from three samples for each point and are presented as the mean ± standard deviation from three independent experiments.

1. Mouse chitotriosidase (Chit1) and acidic mammalian chitinase (AMCase) were expressed in *Escherichia coli* [5,6].
2. Chit1 and AMCase were mixed at molar ratios of 1:1, 1:2 or 2:1 (15:15, 15:30 or 30:15 pmol).
3. The reaction mixtures were incubated with colloidal chitin (final concentration 2 mg/mL) at pH 5.0 or 7.0 and 37 °C for 1 h.
4. The reaction mixture was freeze-dried under vacuum using Centrifugal Concentrator CC-105.
5. The sample was fluorescently labeled by ANTS, separated by PAGE and quantified by the Luminescent Image Analyzer according to the method described above.
6. The molar concentration of the *N*-acetyl-D-glucosamine dimers [(GlcNAc)_2_] by the FACE method was quantified using the standard curve (Fig. 1)

### Preparation of the size marker

1. GlcNAc oligomers (Seikagaku Corporation, Tokyo, Japan) were used as a standard.
2. The oligosaccharide was dissolved in water to a concentration of 1 mM.
3. The standard solution was labeled by the method described above.

### Result and discussion

As previously reported, the improved FACE method can quantify chitooligosaccharides of various sizes that are directly obtained from enzymatic reactions at pH 2.0–8.0 using several buffer systems commonly employed in the biochemical evaluation of chitinolytic activities [3]. Here, we established a standard curve (*R*^2^ = 0.998; *y* = 25.473x + 4.4025) demonstrating the linear association between fluorescence intensity and (GlcNAc)_2_ level. The standard curve warrants quantification of chitooligosaccharides of up to 10 nmol regardless on used buffer system (Fig. 1).

**Fig. 2:**
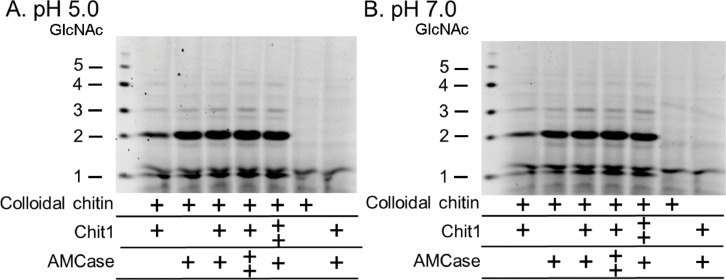
Degradation of colloidal chitin by combining Chit1 and AMCase at pH 5.0 and 7.0. Chitin was incubated with the enzyme mixture at different ratios at pH 5.0 (A) or 7.0 (B).

**Fig. 3:**
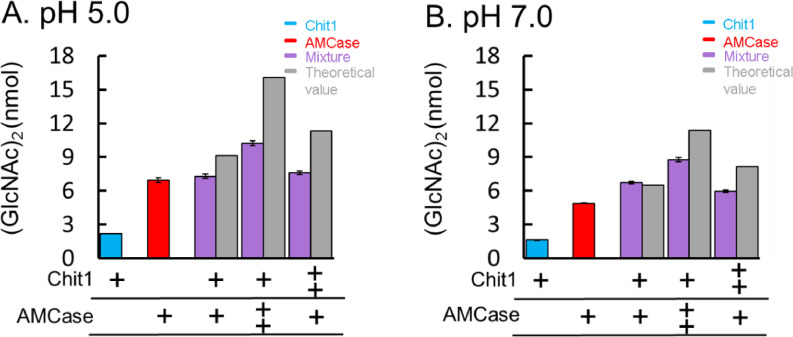
Degradation of colloidal chitin by combining two mammalian chitinases at pH 5.0 and 7.0. Chitin was incubated with the mixture of Chit1 and AMCase at different ratios at pH 5.0 (A) or 7.0 (B). The production value by the single enzymes are shown in blue and red. Chit1 and AMCase were mixed at molar ratios of 1:1, 1:2 or 2:1 (15:15, 15:30 or 30:15 pmol); the mixtures are shown in purple. The theoretical values of the sum of the Chit1 and AMCase activities are shown in gray.

The (GlcNAc)_2_ production efficiency by *Serratia* ChiA and ChiB combined was 2-fold higher when compared with a single enzyme showing that the *Serratia* chitinases act synergistically in chitin degradation [7,8]. Chit1 and AMCase mRNA levels in monocytes and macrophages are responding to cytokines. The expression level of Chit1 in activated macrophages is higher than that of AMCase while lower in lipopolysaccharide (LPS)-treated monocytes [9]. To clarify the mutual effects of Chit1 and AMCase on chitin degradation, we analyzed the degradation products from colloidal chitin by their combination at pH 5.0 or 7.0 and incubation at 37 °C for 1 h. The (GlcNAc)_2_ production efficiency by the combination of the enzymes was lower than that of the calculated sum (theoretical level) of their respective activities at pH 5.0 (Figs. 2A and 3A) while at pH 7.0, the dimer levels were comparable or slightly lower (Figs. 2B and 3B). These results are consistent with the previously reported data using (GlcNAc)_4_ in the condition where large amounts of the substrate remain present [10]. These results suggest that the two enzymes did not compete for the substrates. Our results indicate that, in contrast to the bacterial chitinases, the synergistic effect of mouse enzymes is less pronounced or absent at pH 5.0. At pH 7.0, on Chit1 and AMCase have no synergistic effect and suggest that these enzymes may act independently under various pH conditions [10].

## Acknowledgments

This work was supported by the Project Research Grant from the Research Institute of Science and Technology, Kogakuin University; by Grants-in-Aid for Scientific Research from the Japan Society for the Promotion of Science (JSPS) (grant numbers 19J15483 and 16K07699); by a grant from the Science Research Promotion Fund of the Promotion and Mutual Aid Corporation (PMAC) for Private Schools of Japan; and a grant of the Strategic Research Foundation Grant-aided Project for Private Universities (S1411005) from the Ministry of Education, Culture, Sport, Science and Technology, Japan.

## Conflict of interest

The authors declare no competing interests.
